# An Outbreak of Gastroenteritis Associated with GII.17 *Norovirus*-Contaminated Secondary Water Supply System in Wuhan, China, 2017

**DOI:** 10.1007/s12560-019-09371-7

**Published:** 2019-02-09

**Authors:** Xuan Zhou, De-Guang Kong, Jing Li, Bei-Bei Pang, Ying Zhao, Jun-Bo Zhou, Ting Zhang, Jun-Qiang Xu, Nobumichi Kobayashi, Yuan-Hong Wang

**Affiliations:** 1 Division of Microbiology, Wuhan Centers for Disease Prevention and Control, No. 24 Jianghan North Road, Wuhan, 430015 Hubei People’s Republic of China; 2 Division of Infectious Diseases Control, Wuhan Centers for Disease Prevention and Control, No. 24 Jianghan North Road, Wuhan, 430015 Hubei People’s Republic of China; 3 Institute of Infectious Diseases Control, Hubei Provincial Center for Disease Control and Prevention, No. 6 Zhuodaoquan North Road, Wuhan, 430079 Hubei People’s Republic of China; 4 Department of Hygiene, Sapporo Medical University School of Medicine, S1 W17, Chuo-Ku, Sapporo, 0608556 Japan

**Keywords:** *Norovirus*, Outbreak, Secondary water supply system, Epidemiology, Phylogenetic analysis

## Abstract

A gastroenteritis outbreak occurred in a university in May, 2017, Wuhan, China. The epidemiological survey and pathogen analysis were conducted to identify the pathogen and control this outbreak. Feces or anal swabs from individuals, water, and swabs taken from tap surfaces of the secondary water supply system (SWSS) and foods were collected for the detection of viruses and pathogenic enteric bacteria by real-time RT-PCR and culture, respectively. Nucleotide sequences were determined by RT-PCR and direct sequencing. Genotyping, phylogenetic, and recombination analyses were conducted by a web-based genotyping tool, MEGA, and RDP4 programs, respectively. Of 144 individuals enrolled, 75 met the case definitions. The epidemic curve showed one peak of incidence suggesting the most probable spread of a single common source. In total, 33 specimens were collected before disinfection of the SWSS. Of these, *norovirus* was detected and identified as GII.P17-GII.17 with 100% nucleotide sequence identity among the strains detected in ten students (10/14), a maintenance worker (1/2) dealing with the SWSS, four water samples (4/8), and two swabs taken from tap surfaces (2/3). Pathogens including *Vibrio cholerae*, *Salmonella*, *Shigella, Vibrio parahaemolyticus, Bacillus cereus*, enteropathogenic *Escherichia coli, rotavirus, astrovirus*, and *sapovirus* were negative. The GII.17 strains in this outbreak clustered closely in the same branch of the phylogenetic tree, and slightly apart from the strains of other cities in China, neighboring countries and regions, European and American countries. This gastroenteritis outbreak was deduced to be attributed to GII.P17-GII.17 *norovirus* contamination of the SWSS.

## Introduction

*Noroviruses* belong to the family *Caliciviridae*, genus *Norovirus. Norovirus* is the major pathogen of acute non-bacterial gastroenteritis around the world (Green 2007). The outbreaks of *noroviruses* usually take place in hospitals, nursing homes, schools, and nurseries (Green 2007). The incubation period of *norovirus* gastroenteritis is 24–48 h and the typical symptoms include vomiting, diarrhea, low fever, abdominal pain, and nausea that usually persist 12–60 h (Kaplan et al. 1982). *Norovirus*es are highly infectious as only 10 virus particles can cause infection, and can survive in disinfected environments (Teunis et al. 2008; Leon et al. 2011). Humans are the only known reservoir for human *norovirus*, and transmission occurs by three general routes: person-to-person, foodborne, and waterborne. Person-to-person transmission might occur directly through the fecal–oral route, by ingestion of aerosolized vomitus, or by indirect exposure via fomites or contaminated environmental surfaces (Hall et al. 2011). Although waterborne transmission accounts for 1.5% of *norovirus* outbreaks worldwide, it has been rarely reported in China (Kroneman et al. 2008; Qin et al. 2016; Zhou et al. 2016).

*Norovirus*es are non-enveloped, single-stranded, positive-sense RNA viruses with a genome of approximately 7.5–7.7 kb in length. The genome of human *norovirus* consists of three open reading frames (ORFs) designated ORF1, 2, and 3, of which ORF2 and ORF3 encode the major capsid protein (VP1) and the minor capsid protein (VP2), respectively (Green 2007). Based on the amino acid sequence of VP1, *norovirus*es are classified into at least six genogroups, among which genogroups I, II, and IV are found in humans, with the genogroup II (GII) being predominant. According to the amino acid sequence of capsid and RNA-dependent RNA polymerase (RdRp) gene, GI and GII can be further divided into 14 and 17 genotypes, respectively (Zheng et al. 2006; Kroneman et al. 2013; Vinjé 2015). The new GII.17 variant (Kawasaki variant) emerged as the major cause of *norovirus* gastroenteritis outbreaks in China in late 2014 and spread across four continents (Chan et al. 2017). In this paper, we report a gastroenteritis outbreak associated with a *norovirus* GII.17-contaminated secondary water supply system (SWSS).

From April 28th through May 8th 2017, an acute gastroenteritis outbreak causing cases with a sudden onset of vomiting and diarrhea in a university was reported to the National Notifiable Reportable Diseases Surveillance System and notified to Wuhan Centers for Disease Prevention and Control in Wuhan, Hubei, China. In order to identify the pathogen and control the outbreak, epidemiological and environmental surveys and laboratory detection were conducted.

## Materials and Methods

### Case Definitions

A case was defined as an illness with two or more episodes of vomiting or three or more episodes of diarrhea or one or more episode of both in a 24-h period when non-infectious causes have been ruled out (https://nccid.ca/debrief/norovirus/).

### Epidemiological Investigation

More than half of the cases were enrolled in a risk factor study. The cases and controls were selected randomly from symptomatic and asymptomatic students, respectively, with the ratio of 1:1. Epidemiological information such as the demographics, the prevalence situation, the clinical data, and the potential risk factors was collected by questionnaires from all individuals. The maintenance workers dealing with the SWSS were required to provide the health certificates, logbooks, etc.

### Environmental Investigation

Environmental investigation was conducted to obtain the information including the layout of the building, the distribution of the health facilities, the water supply conditions, sanitation conditions, the disinfection records, and the reports on water quality.

### Specimen Collection

To avoid cross-contamination among specimens, specimen collection and pathogen detection were performed by two institutions. Feces, anal swabs, and the restoring water samples were collected and the pathogens in the specimens were detected by the staff of Wuhan Centers for Disease Prevention and Control. The food specimens, the water samples, and the swabs taken from tap surfaces were collected and the pathogens in the specimens were detected by the staff of Hubei Provincial Center for Disease Control and Prevention (Table 2).

The food specimens including potatoes, pork, fish, bean sprout, sea weed, and *Pleurotus eryngii* were collected from three canteens scattered in the campus of the university where most staff and students used to have meals daily. The peripheral water and the swabs taken from tap surfaces were collected from different rooms where the first two cases and many following cases were found. The water sample of the test center was collected as a control (Table 2).

### Sample Processing

To concentrate viruses from foods, water, and swabs taken from tap surfaces, positively charged microporous filters and ultrafiltration were used for adsorption and elution. The concentration was performed according to Microbiology of Food and Animal Feed-Horizontal Method for Determination of Hepatitis A Virus and *Norovirus* in Food Using Real-time Reverse transcription-Polymerase chain reaction (RT-PCR) (ISO/TS 15216:2013). The assay procedures include the preparation of concentrated suspensions of 10% (wt/vol) stool in PBS.

### RNA Extraction

RNAs of all specimens were extracted by Nucleic Acid Extraction Kit (Jiangsu Tianlong Science & Technology Co., Ltd., Jiangsu, China) according to the manufacturer’s instructions.

#### Free Chlorine Residual Testing and Pathogen Detections

The free chlorine residual in water was detected by using spot rapid determination according to Standards for Drinking Water Quality (GB5749-2006) (Jin et al. 2006).

All samples were cultured for the detection of *V. cholerae*, *Salmonella*, *Shigella, V. parahaemolyticus, B. cereus*, and enteropathogenic *E. coli*.

Group A rotavirus, GI and GII *norovirus*es, *astrovirus*, and *sapovirus* were detected by using Real-time RT-PCR Detection Kits with Cat No. DD-0044-02, DR-0325-02, DR-0150-02, and DR-0164-01, respectively (Shanghai ZJ Bio-Tech Co., Ltd., Shanghai, China), on Life Technologies ABI 7300 fast instrument (de Medici et al. 2004; El-Senousy et al. 2007; Tcheremenskaia et al. 2007; Svraka et al. 2010). The RNA was reverse-transcribed and amplified in a 25 µL reaction mixture containing 1 µL Enzyme Mix, 18 µL Buffer, 1 µL internal control, and 5 µL of RNA. Amplification conditions were as follows: 45 °C for 10 min; 95 °C for 15 min; 40 cycles of 95 °C for 15 s, 60 °C for 60 s, and laser-induced fluorescence detection was on 60 °C.

### RT-PCR and Sequencing

Partial RdRp and capsid protein genes together with the region covering the junction between RdRp and capsid genes, and the whole genomes including complete non-structural polyprotein genes, capsid VP1 and VP2 genes were amplified by RT-PCR using PrimeScript™ One Step RT-PCR Kit Ver.2 (Takara) in a 15 µL reaction mixture consisting of the following: 0.6 µL PrimeScript 1 Step Enzyme Mix, 7.5 µL 2 × 1 Step Buffer, 0.1 µL 100 µM of each primer (JV12 and JV13 for partial region of RdRp, G2SKF, and G2SKR for partial region of capsid), 3.7 µL of TE, and 3 µL of RNA. Primers are listed in Table 1 (Kojima et al. 2002; Vinjé et al. 2003; Wang et al. 2012). Amplification conditions were as follows: 50 °C for 30 min; 94 °C for 2 min; 40 cycles of 94 °C for 30 s, 55 °C for 30 s, 72 °C for 1 min 10 s; and a final extension of 72 °C for 10 min; 4 °C for 5 min. Following 1.5% agarose gel electrophoresis, the PCR products were sent to Sangon Biotech Company for direct sequencing.

**Table 1:** Primers used for RT-PCR and sequencing in this study

Primer	Target gene	Polarity	Sequences(5′–3′)	Nt. position^b^	Purpose	References
JV12W	NSP^a^	+	AYAAGTACCACTATGATGCAG	4284–4304	RT-PCR & sequencing	This study
JV13	NSP	−	TCATCATCACCATAGAAAGAG	4594–4614	RT-PCR	Vinjé et al. (2003))
G2SKF	VP1	+	CNTGGGAGGGCGATCGCAA	5054–5073	RT-PCR	Kojima et al. (2002))
G2SKR	VP1	−	CCRCCNGCATRHCCRTTRTACAT	5376–5398	RT-PCR & sequencing	Kojima et al. (2002))
G2B	VP1	+	TGGAGGGCGATCGCAATCT	5058–5076	RT-PCR & sequencing	Wang et al. (2012))
T17G2SKR	VP1	−	CCACCAGCATACCCATTGTACAT	5376–5398	RT-PCR & sequencing	This study
1F	NSP	+	TGAATGAAGATGGCGTCTAAC	5–22	RT-PCR & sequencing	This study
1R	NSP	−	CGTTGAGGTCTAGGACCCAAC	642–662	RT-PCR & sequencing	This study
2F	NSP	+	GAAATAACACCGCTGTCTCTC	506–526	RT-PCR & sequencing	This study
2R	NSP	−	TATGTGGCCAGGCTGTCTTTAT	1293–1314	RT-PCR & sequencing	This study
3F	NSP	+	CTAACGAACTAGCCATGGTG	1203–1222	RT-PCR & sequencing	This study
3R	NSP	−	GTCTGGTCTGAAATGGTCTTT	1922–1942	RT-PCR & sequencing	This study
4F	NSP	+	TATGCAGACGCACCTGACATT	1859–1879	RT-PCR & sequencing	This study
4R	NSP	−	CCTTAGCAATGGCAAGCTCTTC	2801–2822	RT-PCR & sequencing	This study
5F	NSP	+	GACCTCACTATTGACTCTAG	2603–2622	RT-PCR & sequencing	This study
5R	NSP	−	ATGTATGGACATCCGCAGTCA	3448–3468	RT-PCR & sequencing	This study
6F	NSP	+	TGAAAATCCAAGGTAGAACGG	3357–3377	RT-PCR & sequencing	This study
6R	NSP	−	CCAGTGGGCAATAGAATTCCAT	4501–4522	RT-PCR & sequencing	This study
8F	VP1	+	GACCCCTGGATTAGAACAAAT	5238–5258	RT-PCR & sequencing	This study
8R	VP1	−	ATAGGTTGAAACCCACGCCT	6148–6167	RT-PCR & sequencing	This study
9F	VP1	+	AACGTGACAGGTGGCACATG	5980–5999	RT-PCR & sequencing	This study
9R	VP1	−	AGGGCTATCATTTCAGATTGC	6904–6924	RT-PCR & sequencing	This study
10F	VP2	+	TTCATTGCAGGATTGGCAGGC	6728–6748	RT-PCR & sequencing	This study
10R	VP2	−	GATACAAATTAGCCAAATTTAG	7503–7524	RT-PCR & sequencing	This study

^a^Non-structural polyprotein

^b^Location of the 5′ of the primer in the nucleotide sequence of GZ2015-L339 strain (KT970374)

### Genotyping, Phylogenetic, Recombination, and Statistic Analyses

The genotype of *norovirus* was preliminarily assigned by BLAST (https://blast.ncbi.nlm.nih.gov/Blast.cgi), and confirmed by a web-based genotyping tool (http://www.rivm.nl/mpf/norovirus/typingtool Version2.0) (Kroneman et al. 2011). Phylogenetic analysis was conducted together with reference strain sequences in the Genbank database by MEGA program version 7.0.26 (Kumar et al. 2016). Initial trees for the heuristic search were obtained automatically by applying Neighbor-Join and BioNJ algorithms to a matrix of pairwise distances estimated using the Maximum Composite Likelihood (MCL) approach, and then selecting the topology with superior log likelihood value. The tree was drawn to scale, with branch lengths measured in the number of substitutions per site. Recombination events were analyzed using the Recombination Detection Program (RDP4) (Martin et al. 2015). The GenBank accession numbers of the nucleotide sequences determined in this study are MF421538, MF421551 and MG557567. Statistic analysis was performed by SPSS version 18.0 (SPSS Inc., Chicago, IL, USA) software with a significance level of 0.05 (*P* value).

## Results

### Descriptive Epidemiology

**Fig. 1:**
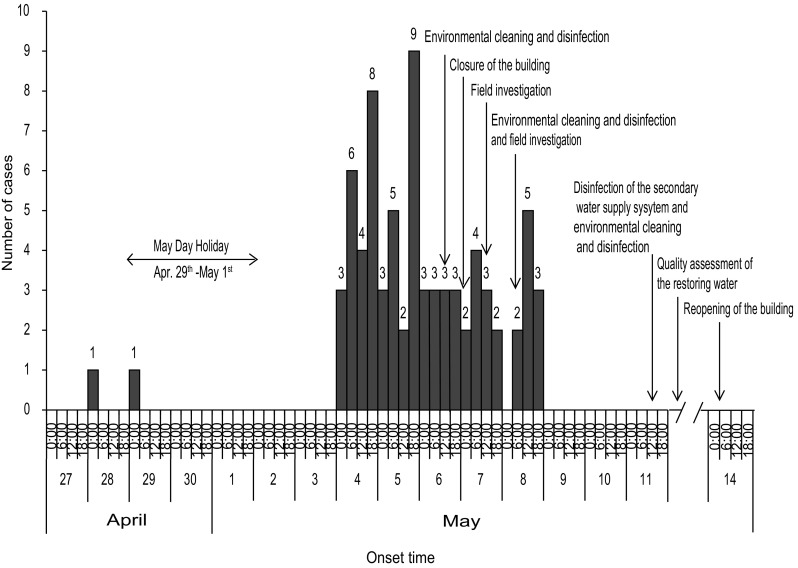
Epidemic curve of acute gastroenteritis cases (*N* = 75) in the outbreak occurred in Wuhan, China, 2017

This outbreak lasted for 11 days (Fig. 1). The first case occurred before dawn on April 28th and followed by the second one on April 29th. No case was reported from April 30th through May 3rd. Then the cases rapidly increased with a peak on May 4th–5th accounting for 53.3% of all cases (40/75). From May 6th through 8th, there were 10–12 cases per day. From May 9th on no case emerged and until May 11th all the cases were recovered.

Of 144 individuals enrolled, 75 met the case definitions and were consisted of 74 students (18–30 year olds) with male-to-female ratio of 1.08:1, while one case was a teacher (female, 48 years old). Of all cases, 69 (92%) were from the School of Life Sciences (SLS) (1 teacher, 54 postgraduates, and 14 undergraduates), the remaining 6 were from the Schools of Information Management (2/6), Foreign Language and Literature (1/6), Economic and Management (1/6), Pharmaceutical Science (1/6), and Resource and Environmental Science (1/6). The staff and the students usually had meals in three canteens, sometimes in restaurants outside of the university or ordered take-away meals online. The students’ dormitories were dispersed over the campus. Symptoms among these 75 patients were typical and consistent with the clinical features of *norovirus* gastroenteritis including diarrhea (72%, 54/75), vomiting (57%, 43/75), nausea (53%, 40/75), abdominal pain (31%, 23/75), fever (21%, 16/75), dizziness (7%, 5/75), and headache (5%, 4/75). The majority had symptoms that were mild while several experienced severe vomiting (up to 8 times /day) and diarrhea (up to 15 times /day). Duration of symptoms was 1–3 days. There was no death case reported.

Further investigation was focused on the SLS. There were 170 teaching and administrative staff, 862 postgraduates, and 580 undergraduates registered in the SLS. During the outbreak, two maintenance workers, most postgraduates and a few undergraduates stayed in the building. The investigation showed that the workers had no health certificate and could not provide their log books. The first two cases were postgraduates who studied in room 3109 with other three postgraduates. On the day before the first case developed the symptoms, she (the case) had had breakfast and lunch, respectively, in two of three canteens mentioned above, while supper together with the other four students in a restaurant near to the university. After supper, they bought pineapple and tomatoes from a fruit store. The pineapple was washed by the staff of the store, while the tomatoes were washed in the lab room by the students.

Although students of other schools had meals in the same three canteens as students of the SLS did, none of them was attacked. The attack rate was much higher in the postgraduates than undergraduates (x^2^ = 10.49, *P* < 0.01). The case–control study indicated that ingesting the fruits washed in the room sink increased the odds of developing gastroenteritis by 7.104 times (95% confidence interval (CI) 2.741–18.415) among the students. Most cases (77.5%) were probably infected due to the contaminated water remained on fruits (Chinese article in press).

### Environmental Investigation

**Fig. 2:**
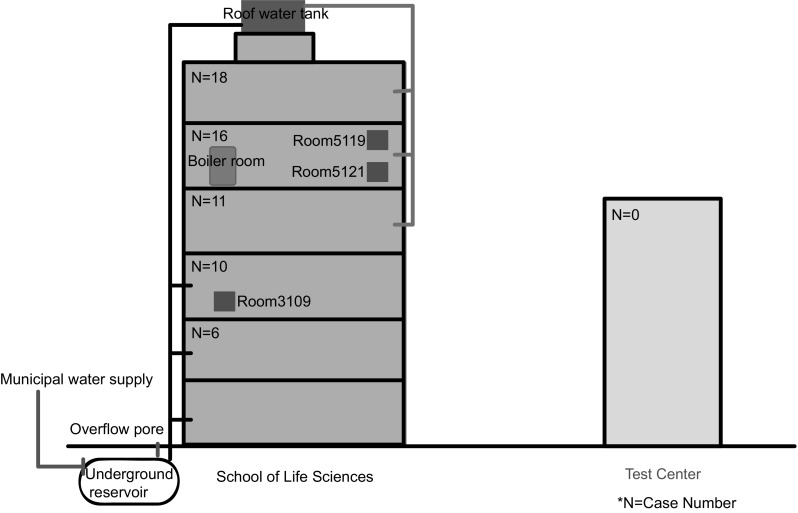
A sketch map for the building of SLS with the labels of the cases distribution, the scope of the SWSS, and the sampling sites

The six-story building of the SLS covers 13,611 square meters, and has more than 80 rooms with poor ventilation. Each floor is equipped with two toilets with liquid soap and paper towels. Drinking water was supplied by the SWSS which covered the whole building. Water from the municipal water supply delivery system was stored in an underground reservoir, and pumped into a water tank on the roof of the building. Water flowed along the pipelines and was supplied to the 1st -3rd and the 4th–6th floor by the reservoir and the water tank, respectively (Fig. 2). The overflow pore of the reservoir was open on the ground outside of the building with weeds surrounded. The overflow pore of the roof tank was unlocked and the outlet port of it missed the stainless steel meshes. None of the test report of water quality, the hygienic license, or the cleaning and disinfection records of the SWSS could be provided.

### Laboratory Detections

A total of 40 specimens were collected including the feces/anal swab, food samples, water samples, and swabs taken from tap surfaces before and after disinfecting the SWSS, respectively (Table 2). Before disinfecting the SWSS, 33 specimens were collected in total. Of these, *V. cholerae*, *Salmonella*, *Shigella, V. parahaemolyticus, B. cereus*, enteropathogenic *E. coli*, *rotavirus*, *astrovirus*, and *sapovirus* were negative, and *norovirus* GII was identified in 11 feces or anal swab samples from 14 students of SLS, and 2 maintenance workers dealing with the SWSS of the SLS. Among the 11 water samples and swabs taken from tap surfaces, *norovirus* GII were detected in six samples. However, *norovirus* was negative in food samples (Table 2). After disinfecting the SWSS, 7 restoring water samples were collected and tested for *norovirus*es with negative results (Table 2). No free chlorine residual had been present in water of the roof tank and the toilet on the 1st floor till May 8th.

**Table 2:** The detection and sequencing of *norovirus* in the acute gastroenteritis outbreak occurred in Wuhan, 2017

Sources	Sample type	Collection date	No. of samples	Detection results (No.)	No. of strains for sequencing		Comment
					Partial RDRP–CAPSID gene	Complete genome	
Student canteens	Food	7 May^b^	6	N	–	–	
Students	Feces	7 May^a^	4	GII(3)	3	1	Symptomatic
Students	Anal swab	8 May^a^	10	GII(7)	7	2	Symptomatic
Maintenance workers	Feces	11 May^a^	2	GII(1)	1	1	Asymptomatic
Room 5119	Sink water	9 May^b^/12 May^a^	1/1	GII(1)/N	1	–	On the 5th floor
Room 5119	Tap surface swab	9 May^b^	1	N			On the 5th floor
Room 5121	Sink water	9 May^b^/12 May^a^	1/1	GII(1)/N	–	–	On the 5th floor
Room 5121	Tap surface swab	9 May^b^	1	GII(1)	–	–	On the 5th floor
Room 3109	Sink water	9 May^b^/12 May^a^	1/1	GII(1)/N	–	–	On the 3rd floor, of the tap
Room 3109	Tap surface swab	9 May^b^	1	GII(1)	–	–	On the 3rd floor, of the tap
Roof water tank	Water	9 May^b^/12 May^a^	1/1	GII(1)/N	–	–	Supply water to the 4th -6th floor
Boiler room	Water (cold)	9 May^b^/12 May^a^	1/1	N/N			On the 5th Floor
Boiler room	Water (hot)	9 May^b^/12 May^a^	1/1	N/N			On the 5th Floor
Test center	Water	9 May^b^	1	N			Opposite to the SLS
Underground reservoir	Water	9 May^b^/12 May^a^	1/1	N/N			Supply water to the 1st -3rd floor
N = Negative							

^a^Samples were collected and the pathogens in the specimens were detected by the staff of Wuhan Centers for Disease Prevention and Control

^b^Samples were collected and the pathogens in the specimens were detected by the staff of Hubei Provincial Center for Disease Control and Prevention

### Genotyping

A total of ten 1057 bp sequences covering partial RdRp and capsid protein genes of *norovirus* were determined in 8 fecal or anal swab samples of 7 students and an asymptomatic worker, and a peripheral water sample from room 5119 in this outbreak, together with feces of a patient (WH2017-36) collected from another outbreak occurred in February in Wuhan (Table 3). The genotype of these 10 strains was identified as GII.P17-GII.17. The strain isolated from water showed 100% nucleotide sequence identity to those of human samples in this outbreak, while WH2017-36 showed 99.8% nucleotide sequence identity to the strains collected in this outbreak (Table 3). The whole genome sequences were determined for four strains collected in this outbreak and one (strain WH2017-36) collected in February. The identity of the nucleotide sequences was 99.9% among the whole genomes of four GII.P17- GII.17 strains collected in this outbreak, while was 99.3–99.6% with the strains collected in China and other countries from 2014 to 2017 (Chan et al. 2015; Chen et al. 2015; Matsushima et al. 2015; Parra and Green 2015; Dang Thanh et al. 2016).

**Table 3:** The nucleotide identity (%) of partial RdRp–capsid gene (1057 bp)

Strains	Collection date	Sample type	Strains																
			WH2017-36	WH2017-151	WH2017-152	WH2017-153	WH2017-156	WH2017-158	WH2017-160	WH2017-162	WH2017-170	WH2017- NoV5121	142,700	ZHITHC-12	CUHK-NS-463	15-AP-1	CAU-267	Kawasaki308	Gaithersburg
WH2017-36	17-Feb-2017	Feces	100	99.8	99.8	99.8	99.8	99.8	99.8	99.8	99.8	99.8	99.7	99.8	99.9	99.9	99.7	99.5	99.7
WH2017-151	8-May-2017	Feces	99.8	100	100	100	100	100	100	100	100	100	99.7	99.8	99.9	99.9	99.7	99.5	99.7
WH2017-152	8-May-2017	Feces	99.8	100	100	100	100	100	100	100	100	100	99.7	99.8	99.9	99.9	99.7	99.5	99.7
WH2017-153	8-May-2017	Feces	99.8	100	100	100	100	100	100	100	100	100	99.7	99.8	99.9	99.9	99.7	99.5	99.7
WH2017-156	8-May-2017	Feces	99.8	100	100	100	100	100	100	100	100	100	99.7	99.8	99.9	99.9	99.7	99.5	99.7
WH2017-158	8-May-2017	Anal swabs	99.8	100	100	100	100	100	100	100	100	100	99.7	99.8	99.9	99.9	99.7	99.5	99.7
WH2017-160	8-May-2017	Anal swabs	99.8	100	100	100	100	100	100	100	100	100	99.7	99.8	99.9	99.9	99.7	99.5	99.7
WH2017-162	8-May-2017	Anal swabs	99.8	100	100	100	100	100	100	100	100	100	99.7	99.8	99.9	99.9	99.7	99.5	99.7
WH2017-170	11-May-2017	Feces	99.8	100	100	100	100	100	100	100	100	100	99.7	99.8	99.9	99.9	99.7	99.5	99.7
WH2017- NoV5121	8-May-2017	Water	99.8	100	100	100	100	100	100	100	100	100	99.7	99.8	99.9	99.9	99.7	99.5	99.7
142,700	2014	No data	99.7	99.7	99.7	99.7	99.7	99.7	99.7	99.7	99.7	99.7	100	99.7	99.8	99.8	99.6	99.4	99.6
ZHITHC-12	2015	No data	99.8	99.8	99.8	99.8	99.8	99.8	99.8	99.8	99.8	99.8	99.7	100	99.9	99.9	99.7	99.5	99.7
CUHK- NS-463	5-Dec-2014	No data	99.9	99.9	99.9	99.9	99.9	99.9	99.9	99.9	99.9	99.9	99.8	99.9	100	100	99.8	99.6	99.8
15-AP-1	Feb-2015	Feces	99.9	99.9	99.9	99.9	99.9	99.9	99.9	99.9	99.9	99.9	99.8	99.9	100	100	99.8	99.6	99.8
CAU-267	2015	Feces	99.7	99.7	99.7	99.7	99.7	99.7	99.7	99.7	99.7	99.7	99.6	99.7	99.8	99.8	100	99.4	99.6
Kawasaki308	2015	Feces	99.5	99.5	99.5	99.5	99.5	99.5	99.5	99.5	99.5	99.5	99.4	99.5	99.6	99.6	99.4	100	99.4
Gaithersburg	25-Nov-2014	Feces	99.7	99.7	99.7	99.7	99.7	99.7	99.7	99.7	99.7	99.7	99.6	99.7	99.8	99.8	99.6	99.4	100

### Phylogenetic and Recombination Analyses

**Fig. 3:**
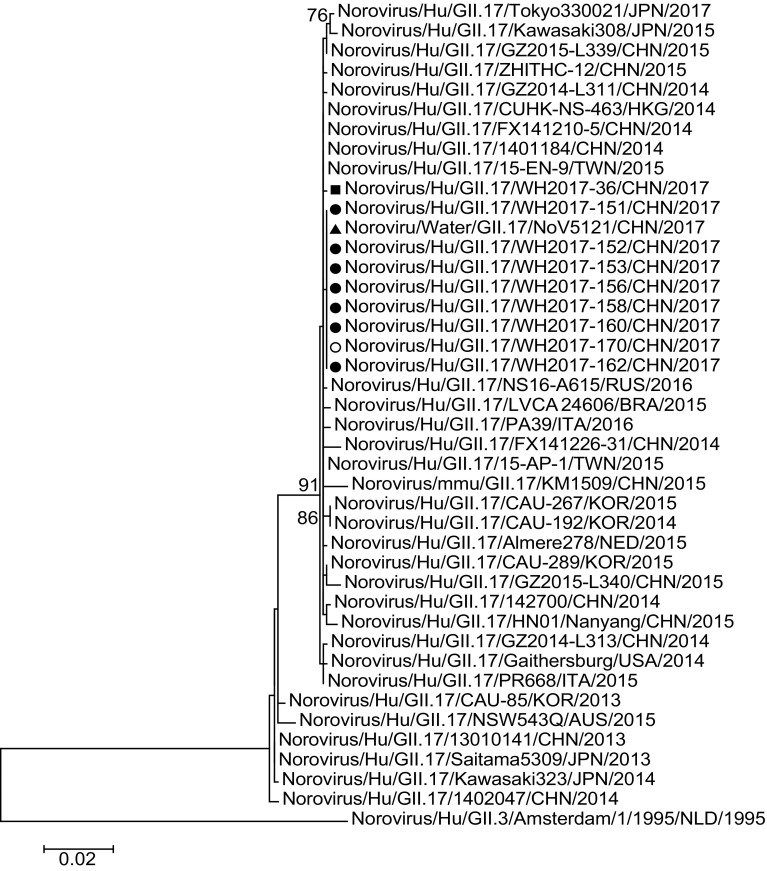
Phylogenetic tree of partial RDRP–CAPSID gene of *norovirus* GII.17 (1057 bp). The strains obtained in the outbreaks in Wuhan, China, 2017, were marked by caret shapes. Filled square indicate the strain collected in the outbreak occurred in February of 2017. Filled triangle, open circle and filled circle indicate the strains collected in this outbreak from the water, the asymptomatic worker, and the patients, respectively. The evolutionary history was inferred by using the Maximum Likelihood method based on the Kimura 2-parameter model. The bootstrap values generated from 1000 replicates are shown at nodes, and only bootstrap values ≥ 70% are presented

Phylogenetic tree was constructed based on 1057-bp sequences of partial RdRp capsid gene of 10 GII.17 strains collected in outbreaks in Wuhan and 32 reference strains (Fig. 3). The strains of this outbreak clustered together with the strains identified from 2014 to 2017 in central and southeastern China (Henan, Guangdong, Shanghai, and Nanjing), surrounding nations and regions (Hong Kong, Taiwan, Japan, Korea, and Russia), European countries (Italy, Netherlands), and American countries (Brazil, USA) (Chan et al. 2015; Chen et al. 2015; Matsushima et al. 2015; Parra and Green 2015; Dang Thanh et al. 2016) (Fig. 3). Recombination events were analyzed, but none was found among these 1057 bp sequences of *norovirus* GII.17 strains.

### Control Measures

Comprehensive measures were adopted to control this outbreak. The cases were timely reported and treated. Environmental cleaning and disinfection were executed both on the 6th and 8th of May. The building was closed from May 7th on. A professional disinfection company flushed and disinfected the SWSS including the water tank, the reservoir, and the pipelines, and replaced the water stored in the system on May 11th (Fig. 1). The total area disinfected was more than 23,000 m^2^. A certificate of water quality was provided by a third-party specialist agency entrusted by the company. Moreover, the restoring water samples were collected on May 12th and detected as *norovirus*-negative on May 13th (Table 2). The building was reopened at the night of May 14th since the water quality of the restoring SWSS was double guaranteed (Fig. 1).

## Discussion

This study describes an epidemiological survey and pathogen analysis of a *norovirus* outbreak. The clinical symptoms of the individuals meet the case definitions of *norovirus* gastroenteritis outbreak. Considering that the students had meals in the same three canteens but only the ones from the SLS were attacked, a foodborne outbreak could be preliminary excluded. The analyzed food samples were negative for both enteric pathogenic bacteria and viruses which led to a further exclusion of a foodborne outbreak.

Since the students’ dormitories were scattered throughout the campus but only the ones from the SLS were attacked, the infection source could be suspected in the SLS building. The epidemic curve with one peak of incidence suggested that a single, common infection source could have been responsible for the outbreak (Fig. 1). The Labor Day holiday took place from April 29th through May 1st in China. After that, a morbidity peak appeared and lasted for 2 days with accumulated cases. Although the patients had been isolated and treated, and the building environment including the air, water, surfaces, floors, and walls had been cleaned and disinfected, the morbidity was relatively steady and high during May 6th and 8th. It indicated that the source of infection existed persistently (Fig. 1). In the SLS, the minority of the undergraduates and the majority of the postgraduates were required to participate in daily experimental study in the building. The attack rates in these two groups were consistent with their disparities in the exposure period to the infection source. After the building had been closed and passed an average incubation period, no new case emerged which indicated the blocking of the infection source. The building was reopened after flushing and disinfection of the SWSS, and no new case was reported which verified the removal of the infection source (Fig. 1). Based on the above facts, the outbreak was strongly suspected as a waterborne one and the infection source was certainly in the SLS building.

*Norovirus* is a chlorine-resistant virus, and 20 ppm chlorine causes no significant reduction in human *norovirus* infectivity. According to Standards for Drinking Water Quality (GB5749-2006) of China, the lower limit of the free chlorine for drinking water is 0.05 mg/L (Jin et al. 2006). The water in the roof tank and the peripheral water of the toilet on the 1st floor were insecure with no free chlorine residual presence to safeguard the water from *norovirus* contamination. The overflow pores of the underground reservoir and the roof water tank were open, unlocked, and easy to be contaminated by pathogens through contacts. The water sample collected from the roof tank was positive for *norovirus* that indicated the presence of *norovirus* contamination. Although the water supplied to the 1^st^–3rd floor by the underground reservoir was negative for *norovirus*, 16 cases developed symptoms. *Norovirus*es are highly infectious as only 10 virus particles can cause infection, and can remain infectious in ground water even after storage at room temperature for at least 61 days (Teunis et al. 2008; Seitz et al. 2011). It could be deduced that the contaminated SWSS was likely to be responsible for this outbreak.

The contaminated water was the vital risk factor since the odds ratio of developing gastroenteritis was 7.104 in the case–control study. The probable infection pathway of most cases was ingestion of remaining *norovirus*-contaminated water on the fruits. The pathogen of the outbreak was further confirmed as the same GII.17 *norovirus* due to the 100% identity among the partial RdRp–capsid genes (1057 bp) of the students, the maintenance worker, and the peripheral water sample (Table 3; Fig. 3). Considering that the water sample from the test center was negative for pathogens and no case of gastroenteritis was reported from that building, the detection results and the identification analysis of the pathogens indicated that there was a very close relationship between the *norovirus* outbreak and the contaminated SWSS of the SLS.

The strains obtained in this outbreak clustered together in the same branch of the phylogenetic tree. Strain WH2017-36 was aside although it was of the same genotype and was collected in the same year (Fig. 3). The characteristics of emergence, geographical spread, and evolution of GII.17 genotype *norovirus*es have been increasingly focused on (de Graaf et al. 2015). Viruses of the GII.17 genotype have been circulating in the human population for at least 40 years since the first GII.17 strain reported in 1978 (Rackoff et al. 2013). GII.17 cases were sporadically reported worldwide such as in Africa, Asia, Europe, and America (Verhoef et al. 2015). More widespread circulation of GII.17 was first reported for environmental samples in Korea from 2004 to 2006 (Dang Thanh et al. 2016). However, the waterborne outbreaks caused by GII.17 *norovirus* are quite limited in the world (Arvelo et al. 2012; Qin et al. 2016). In the winter of 2014/15, genetically closely related GII.17 viruses were first detected in gastroenteritis outbreaks in the Guangdong province, and soon increased in outbreaks during the same winter in Shanghai, Jiangsu, and other provinces in China (Chen et al. 2015; Fu et al. 2015). The novel GII.17 variant was also the predominant genotype in surrounding nations and regions during the same period of time (Matsushima et al. 2015). Previously, *norovirus* GII.17 was detected in sporadic gastroenteritis in Wuhan (Wang et al. 2012). A total of 27 *norovirus* outbreaks occurred in Wuhan in 2017. Of these, *norovirus* GII.P16 / GII.2 was the predominant pathogen (data not shown). Only two outbreaks were caused by *norovirus* GII.17; one occurred in February (WH2017-36) and the other in May reported in this study. Although the dominant genotype of *norovirus* outbreaks has shifted from new GII.17 Kawasaki variant to GII.P16 / GII.2 in China, the GII.17 *norovirus* outbreaks still occurred occasionally (Ao et al. 2017; Qin et al. 2017).

Closure of the building and disinfection of the SWSS were efficient in controlling the outbreak. Taking into consideration of the finding of genetic analysis, the results of epidemiological investigation and environmental survey, and the pathogens detection, the *norovirus*-contaminated SWSS was suggested to be closely related to this outbreak. A few details of exact infection source still remained unclear. The asymptomatic maintenance worker admitted washing his hands in the toilet sink but denied having contacted the roof tank water directly. The water supply network wiring diagrams of the SWSS were not exact because of multiple adaptations.

Waterborne *norovirus* outbreaks were reported in the world, and the majority of them were linked to the water bodies, the groundwater, and sewage treatment systems, etc (Kauppinen et al. 2017; Polkowska et al. 2018). It is worth noting worldwide that very few waterborne *norovirus* outbreaks linked to the municipal water supply systems and the SWSS were reported including seven representative studies in Italy, Sweden, United States, and China (Riera-Montes et al. 2011; Li et al. 2013; Giammanco et al. 2018). In these seven literatures, the patients were *norovirus*-positive in all reported outbreaks. However, only of three outbreaks, the pathogen in the water samples was identified as *norovirus*. Of the rest four outbreaks, the presence of bacterial indicators of fecal contamination or the coliphages marker of viral contaminations was identified instead of the pathogens. The drinking water was contaminated by different pathways such as broken pipeline, sewage leakage, cross-connections with contaminated industrial water system, and contamination of the well and springs. These indicate that the management of water supply systems is important and should not be ignored.

In China, the buildings of six or more floors supply pressurized water by the SWSS, which is a challenge unique to China comparing with developed nations. The health certificate of the maintenance workers, the working logbooks, flushing, disinfection records of the SWSS, the test reports of the environment, and water quality were not available in this outbreak, which revealed the lack of effective management in the SWSS. Our study reminds that more attention should be paid to the SWSS and suggested that the management and hygienic monitoring on the system should be strengthened. The SWSS should be flushed and disinfected periodically. It is necessary to provide professional training to the staff that are responsible for the SWSS and adequate free residual chlorine should be guaranteed in the storage water. Up till now, only one waterborne *norovirus* outbreak associated with the SWSS was reported globally (Li et al. 2013). The present study was the first report of gastroenteritis outbreak associated with the GII.17 *norovirus*-contaminated SWSS worldwide.
